# MDM2 in Tumor Biology and Cancer Therapy: A Review of Current Clinical Trials

**DOI:** 10.3390/ijms27010099

**Published:** 2025-12-22

**Authors:** Francesco Russano, Mattia Sturlese, Luigi Dall’Olmo, Francesco Callegarin, Davide Brugnolo, Paolo Del Fiore, Vittoria Patti, Arianna Purpura, Stefano Moro, Marco Rastrelli, Simone Mocellin

**Affiliations:** 1 Soft-Tissue, Peritoneum and Melanoma Surgical Oncology Unit, Veneto Institute of Oncology (IOV), 35128 Padua, Italy; francesco.russano@iov.veneto.it (F.R.); davide.brugnolo@iov.veneto.it (D.B.); paolo.delfiore@iov.veneto.it (P.D.F.); vittoria.patti@iov.veneto.it (V.P.); arianna.purpura@iov.veneto.it (A.P.); marco.rastrelli@unipd.it (M.R.); simone.mocellin@unipd.it (S.M.); 2 Molecular Modeling Section, Department of Pharmaceutical and Pharmacological Sciences, University of Padua, 35131 Padua, Italy; mattia.sturlese@unipd.it (M.S.); stefano.moro@unipd.it (S.M.); 3 Department of Surgery, Oncology and Gastroenterology (DISCOG), University of Padua, 35128 Padua, Italy; 4 Clinical Research Unit, Veneto Institute of Oncology IOV-IRCCS, 35128 Padua, Italy; francesco.callegarin@iov.veneto.it

**Keywords:** MDM2, p53, MDM2 inhibitors, cancer therapy, drug development, clinical trial

## Abstract

The Murine Double Minute 2 (*MDM2*) gene encodes an E3 ubiquitin ligase that negatively regulates the tumor suppressor p53, maintaining low p53 levels through ubiquitination and proteasomal degradation. MDM2 overexpression in various malignancies leads to reduced p53 activity, contributing to tumor initiation and resistance to therapies. As such, MDM2 is a promising target for drug development. Innovative small-molecule inhibitors are being designed to disrupt the MDM2-p53 interaction, thereby restoring p53’s tumor-suppressive functions. This review focuses on clinical trials evaluating MDM2 inhibition for cancer therapy. MDM2 exerts its oncogenic effects primarily through its interaction with p53 but also has p53-independent functions involved in cell cycle progression and DNA repair. Elevated MDM2 expression is associated with poor prognosis across various cancers, including dedifferentiated liposarcoma, breast cancer, and glioblastoma. Targeting MDM2 with inhibitors has shown promising potential in clinical development, aiming to reactivate p53’s functions in tumors with wild-type TP53, improving therapeutic outcomes in cancer treatment.

## 1. Introduction

The Murine Double Minute 2 (*MDM2*) gene encodes an E3 ubiquitin ligase that serves as a principal negative regulator of the tumor suppressor p53. Under physiological conditions, MDM2 maintains low intracellular levels of p53 by mediating its ubiquitination and subsequent proteasomal degradation, thereby preventing inappropriate activation of apoptosis, cell cycle arrest, or senescence [1]. In various malignancies, MDM2 is frequently overexpressed, leading to excessive suppression of p53 activity. The p53-MDM2 association contributes significantly to tumor initiation, progression, and therapeutic resistance [2,3]. Given its central oncogenic role, MDM2 has emerged as a compelling target for anticancer drug development [4]. Innovative small-molecule inhibitors targeting the MDM2-p53 interaction are being developed to restore p53 function in tumors that harbor wild-type TP53. By disrupting this interaction, these inhibitors carry the potential to reactivate p53’s tumor-suppressive capabilities, offering a promising therapeutic strategy. This manuscript provides a comprehensive analysis of MDM2 in oncology, evaluating the current landscape and prospects of MDM2-targeted therapies.

## 2. Biological Role of MDM2

### 2.1. p53-Mediated Oncogenic Regulation

MDM2 exerts its primary oncogenic effect through interaction with p53. It binds to the N-terminal transactivation domain of p53, suppressing its transcriptional activity and promoting its degradation via the ubiquitin–proteasome system. This interaction forms an autoregulatory loop where p53 induces MDM2 expression, which in turn downregulates p53 activity. What proved to be extremely interesting in this relation is the phosphorylation mechanism that can disrupt the interaction with p53, modifying several states, including stability, oligomerization, and subcellular localization [1,5].

This tightly regulated mechanism is crucial for homeostasis, which is disrupted in tumors by MDM2 overexpression or gene amplification. In 2004, Vassilev and his team published a study revealing how potent small-molecule antagonists of MDM2 function. Their analysis of crystal structures showed that these compounds bind to the p53-binding pocket of MDM2, activating the p53 pathway in cancer cells. This leads to cell cycle arrest, apoptosis, and significant growth inhibition of human tumor xenografts in nude mice, paving the way for novel cancer therapies targeting MDM2 [6].

### 2.2. p53-Independent Functions

It has been reported that MDM2 also possesses p53-independent oncogenic activities. In particular, it binds to other regulators such as p73, E2F1, Nbs1, and Rb, modulating cell cycle progression, DNA repair, and apoptosis independently of p53. MDM2’s interaction with Nbs1 impairs homologous recombination, increasing genomic instability, interfering with DNA repair, and promoting aberrant chromosomal events that facilitate neoplastic transformation and tumour progression [7]. MDM2 stimulates the transcriptional activity of E2F1/DP-119. E2F1, along with its partner DP-1, drives the cell cycle progression from G1 to S phase. Ablation or inhibition of MDM2 results in reduced levels of E2F1 protein and mRNA. The regulation of TAp73 transcriptional activity occurs through a synergistic cooperation between MDM2 and E2F11. Overexpression of MDM2 alone fails to enhance TAp73 transcriptional activity, but co-overexpression of MDM2 and E2F1 strongly enhances it (up to about 5 times compared to E2F1 alone). This synergistic effect likely involves their physical interaction in the nuclei. MDM2 interacts also directly with the tumor suppressor protein Rb. The MDM2 acidic domain (residues 254 to 264) binds to the C-terminal region of Rb (residues 785 to 803) [8,9]. MDM2 promotes Rb degradation through two distinct mechanisms:

1. Ubiquitin-dependent degradation (observed upon MDM2 overexpression).
2. Ubiquitin-independent proteasomal degradation. In this pathway, MDM2 enhances the interaction between Rb and the C8 subunit of the 20S proteasome.

This creates a critical vulnerability for current therapeutics. Clinical-stage MDM2 inhibitors, such as Nutlin-3a, Milademetan, and Brigimadlin, are designed as “p53-mimetics”: they competitively bind to the N-terminal p53-binding hydrophobic pocket of MDM2. Because these small molecules are highly specific to the hydrophobic pocket, they do not sterically occlude the acidic domain responsible for Rb binding. Consequently, a “functional bypass” of the G1/S checkpoint can occur. Although the inhibitor successfully displaces p53—leading to p53 stabilization and the subsequent induction of p21 (CDKN1A)—this may fail to arrest the cell cycle. p21 normally functions by inhibiting CDKs to maintain Rb in a hypophosphorylated, growth-suppressive state. However, if MDM2 amplification is present, the drug-insensitive acidic domain continues to mediate the physical degradation of Rb. The depletion of Rb protein levels renders the phosphorylation status irrelevant; E2F1 is released, and the cell progresses into S-phase despite the restoration of the p53 transcriptional program. This suggests that in tumors where MDM2-mediated Rb degradation is a dominant driver, blocking the p53 pocket alone is insufficient, highlighting the need for therapeutic strategies that degrade the entire MDM2 protein (e.g., PROTACs) rather than merely inhibiting its p53-binding site [9].

While its role is as an oncogene, through not only the suppression of p53 function remains well established, emerging evidence indicates a possibility that MDM2 may act as a tumor suppressor under certain contexts. MDM2 targets Cadherins, specifically E-cadherin, which plays a critical role in the metastatic process of solid tumors of epithelial origin [1,10].

### 2.3. Regulation of MDM2 Activity

MDM2 activity is tightly regulated through a complex network of post-translational modifications (PTMs) and protein–protein interactions that dictate its stability, localization, and E3 ubiquitin ligase function. Beyond the transcriptional feedback loop with p53, phosphorylation plays a pivotal role in modulating MDM2 activity. Specifically, the AKT/mTOR signaling pathway can induce AKT-mediated phosphorylation and stimulation of MDM2, which promotes p53 inhibition and tumorigenesis. Consequently, targeting this crosstalk through the combined inhibition of AKT/mTOR and MDM2 has emerged as a promising therapeutic strategy in cancers exhibiting excessive activation of this pathway. However, MDM2 phosphorylation is not exclusively dependent on the PI3K/AKT axis; recent evidence demonstrates that the RAS/MAPK pathway is also a critical driver of MDM2 stability. Specifically, downstream effectors of the MAPK pathway, such as p90RSK, have been shown to directly phosphorylate MDM2 at Ser166—a residue classically attributed to AKT activity. This phosphorylation promotes MDM2 nuclear translocation and enhances its E3 ubiquitin ligase activity toward p53, thereby suppressing the tumor suppressor even in the absence of AKT hyperactivation. This mechanism is particularly relevant in tumors with oncogenic upregulation of the MAPK pathway, such as melanoma and lung cancer, where p90RSK inhibition has been observed to restore p53 levels [11]. Furthermore, MDM2’s ubiquitin ligase activity is significantly regulated by its interaction with MDMX (MDM4). While MDMX lacks intrinsic ligase activity, it forms heterodimers with MDM2 that stabilize the protein and enhance its E3 ligase activity; evidence suggests that the MDM2/MDMX heterodimer is a more efficient ubiquitin ligase for p53 compared to the MDM2 homodimer. Conversely, the tumour suppressor ARF (p14ARF) acts as a potent inhibitor not only by sequestering MDM2 in the nucleolus but also by directly inhibiting MDM2’s ubiquitin ligase activity, thereby preventing p53 degradation. Finally, genetic variations such as Single Nucleotide Polymorphisms (SNPs) in the MDM2 promoter (e.g., SNP309T>G) can disrupt the coordinated p53-MDM2 oscillation by increasing MDM2 expression, further influencing cancer susceptibility and the efficacy of therapeutic interventions [12,13].

### 2.4. Implications in Tumor Biology of Specific Cancer Histotypes

MDM2 gene amplification or protein overexpression has been reported in a range of human malignancies, including dedifferentiated liposarcoma (DDLPS), breast cancer, glioblastoma, colorectal cancer, non-small-cell lung cancer (NSCLC), and urothelial carcinoma among others [14]. In many cases, high MDM2 expression correlates with poor prognosis and resistance to chemotherapy or immunotherapy. Therefore, MDM2 has been the target of development for many synthetic small molecules, peptide- and aptamer-based therapies [6,15].

DDLPS in particular serves as the prototypical model of MDM2-driven cancer. MDM2 amplification is particularly common in sarcomas. Among them, liposarcoma is the most frequent histological type of soft-tissue sarcoma, including DDLPS as one of the most frequent. Nearly all DDLPS tumors harbor high-level MDM2 amplification, making it a pathognomonic biomarker and an actionable therapeutic target [16].

### 2.5. MDM2 Inhibitors

#### 2.5.1. Mechanisms of Action and Clinical Development

Targeting the MDM2–p53 interaction is a rational strategy to reactivate p53 functions (e.g., apoptosis and cell cycle arrest) in tumors with wild-type TP53. MDM2 inhibitors bind to the p53-binding pocket on MDM2, preventing ubiquitination and proteasomal degradation of p53 [17].

**Figure 1:**
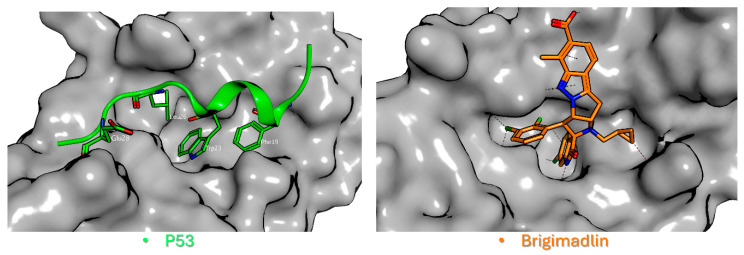
Structural basis of p53-MDM2 protein–protein interaction inhibition. (**Left**): The interaction between p53 (green) and MDM2 (gray) highlights the native binding mechanism (PDB ID: 1YCR) [15]. The p53 α-helix inserts into the MDM2 cleft, with three critical hydrophobic residues (Phe19, Trp23, and Leu26) and one polar Glu28 residue anchoring the peptide. (**Right**): Brigimadlin (orange), a clinical-stage MDM2 inhibitor, binds competitively to the same hydrophobic cleft on MDM2 (gray), sterically blocking p53 association. The compound mimics the helical p53 interface, exploiting hydrophobic contacts (e.g., Trp23 pocket) to disrupt MDM2’s negative regulation of p53 (PDB ID: 8PWC) [30].

From a structural perspective, p53 adopts an α-helical conformation upon binding to MDM2, positioning three critical hydrophobic residues, Phe19, Trp23, and Leu26, into complementary pockets of the MDM2 cleft [15] (Figure 1, left). These pockets serve as the primary binding sites for clinical-stage MDM2 inhibitors, most of which function as competitive protein–protein interaction (PPI) inhibitors by mimicking the p53 α-helix (Figure 1, right).

Notably, while the native p53 peptide exhibits only modest (low micromolar) affinity for MDM2 [18], synthetic inhibitors like Nutlin-3a, SAR405838, Brigimadlin, and ALRN-6924 achieve low-nanomolar or sub-nanomolar binding affinity; a > 1000-fold improvement, obtained by strategic optimization of hydrophobic contacts and additional hydrogen-bond interactions. Before making some observations on clinical progress in terms of MDM2 inhibitors, it is important to specify that the effect of p53 activation by an MDM2 inhibitor in healthy tissue is of immense interest from a toxicology and therapeutic perspective. Several preclinical studies highlight the reduced toxicity of these molecules. Despite this, the precise mechanism for the lack of toxicity of MDM2 inhibitors to normal tissue still needs to be clarified.

Currently, clinical trials on the development of MDM2 inhibitors for cancer therapy are evaluating various compounds. A brief description of the main results addressing monotherapy is provided below.

MI-77301 (SAR405838) is a spiro-oxindole compound with high selectivity for MDM2. Preclinical data support its efficacy in liposarcoma and other p53-wild-type malignancies [19].

Idasanutlin (RG7388) is a second-generation Nutlin with a pyrrolidine structure, for oral use with improved pharmacokinetics. A multicenter, randomized, double-blind phase III trial (MIRROS) in AML integrates Phase II safety and efficacy criteria into a Phase III study via a blinded interim analysis for futility [20]. In this trial the myelosuppressive effect appears to be a limitation. It remains to evaluate if dose changing or different treatment regimens could reduce neutropenia improving efficacy [21].

BI 907828 (Brigimadlin) is an oral MDM2-p53 antagonist [22], currently under evaluation for advanced DDLPS in the Brightline-1 Phase II/III trial (NCT05218499). Early data suggest improved tolerability and disease control [5]. A subsequent trial (Brightline-2 Phase II/III trial -NCT03449381) is evaluating Brigimadlin as a second-line treatment for patients with advanced or metastatic cases of several cancers that often have limited treatment options and poor outcomes. These include Biliary tract cancer (BTC), Pancreatic ductal adenocarcinoma (PDAC), Lung adenocarcinoma, Bladder cancer [23].

Nutlin-3 belongs to the family of Nutlins, identified as cis-imidazoline inhibitors. In particular, the first small-molecule MDM2 inhibitor developed, Nutlin-3, competitively inhibits the p53-binding site of MDM2. It demonstrated robust preclinical efficacy in p53 wild-type models. Otherwise, Nutlin-3 requires functional p53 and MDM2 for its biological activity. The significant efficacy of Nutlin-3, demonstrated in several pre-clinical studies even in tumors with standard MDM2 expression levels, indicates that a broad patient population with wild-type p53 tumors may be responsive to antagonists of the p53–MDM2 pathway [19].

RITA (reactivation of p53 and induction of tumor cell apoptosis) binds directly to p53 and induces a conformational change that prevents MDM2 binding. In particular, RITA leads to the accumulation of p53 through an extension of its half-life. It is also reported that RITA shows p53-independent functions. The structural features identified in p53 and RITA may serve as a blueprint for the design of novel allosteric p53 activators with potential for clinical translation. Although it has a unique mechanism, poor solubility and stability have limited its clinical development until now [24].

#### 2.5.2. Comparative Analysis of MDM2 Inhibitors: Dosing, Toxicity, and Efficacy

Rather than viewing MDM2 inhibitors as a monolithic class, clinical data reveals distinct profiles based on dosing strategies and toxicity management.

Continuous vs. Intermittent Dosing: Early clinical efforts, such as the Phase III MIRROS trial of Idasanutlin in AML, utilized a daily dosing schedule (days 1–5). While this achieved a higher Overall Response Rate (38.8%) compared to placebo, it was associated with severe gastrointestinal toxicity (87% diarrhea) and high rates of febrile neutropenia (52.8%), ultimately failing to improve Overall Survival. Conversely, newer generation inhibitors like Milademetan and Brigimadlin have pivoted to intermittent “pulsed” dosing (e.g., 3 days on/11 days off) [20]. In the Phase II MANTRA-2 trial, this strategy reduced gastrointestinal toxicity but did not eliminate Grade 3/4 thrombocytopenia (25%), highlighting that hematologic toxicity remains a class effect linked to the functional p53 activation in normal hematopoietic progenitors [25].

Efficacy across Indications: The clinical benefit varies significantly by histology. In dedifferentiated liposarcoma (DDLPS), a tumor type driven by MDM2 amplification, efficacy has been modest but reproducible. Milademetan achieved a confirmed ORR of 3.2% (with unconfirmed responses up to 19.4%), whereas Alrizomadlin demonstrated an ORR of 25% specifically in MDM2-amplified, TP53-wild-type solid tumors, with some patients experiencing delayed tumor regression consistent with immunomodulatory activity [26].

Emerging Resistance Patterns: A comparative look at post-treatment data reveals common failure modes. Both the MANTRA-2 and MIRROS trials suggest that resistance is often acquired through the emergence of de novo TP53 mutations or the selection of subclones with upregulated downstream anti-apoptotic factors, underscoring the necessity of combination strategies rather than monotherapy. Table 1 reflects results in terms of Objective Response Rate (ORR), Progression-Free Survival (PFS) or equivalent and incidence of haematological adverse events (AEs) in main clinical trials exploring MDM2-inhibitors [20,25,26].

### 2.6. Combination Strategies and Novel Approaches

#### 2.6.1. Combination Strategies 

Due to the development of resistance and the occurrence of incomplete responses to monotherapy, combination therapy approaches represent a promising alternative for further investigation. These strategies aim to enhance treatment efficacy by targeting multiple pathways simultaneously. Various methods have been explored to achieve improved patient outcomes.

##### Chemotherapy

MDM2 inhibitors can sensitize tumor cells to genotoxic agents such as doxorubicin or cytarabine by reinforcing the DNA damage response through p53 reactivation with a synergic effect [27].

Over time, numerous pieces of evidence have emerged regarding the ability of a combination strategy to activate p53, reduce proliferation and increase the vulnerability of cells exposed to chemotherapy. In neuroblastoma, it has been shown that the combination of Nutlin-3a with etoposide or cisplatin significantly reduces proliferation. Furthermore, Nutlin-3a has been shown to inhibit the functioning of ABC transporters, P-glycoprotein and multidrug resistance protein 1 (MRP1; ABCC1) [28].

##### Immunotherapy

Recently, Zeng and collaborators conducted a comprehensive review highlighting the crucial role of MDM2 in shaping the immune microenvironment, facilitating tumor immune evasion, and driving hyperprogression during immunotherapy.

Reactivation of p53 may enhance tumor immunogenicity, thereby increasing the efficacy of checkpoint inhibitors. Trials are ongoing for combinations with anti-PD-1/PD-L1 antibodies, especially in NSCLC and melanoma [29]. In particular, by blocking MDM2, these drugs can help re-sensitize tumors to the effects of immunotherapy, especially in malignancies that have high levels of MDM2, as a result of overexpression or amplification. It has also been shown a crucial role in T-cell regulation for MDM2 and MDM2 inhibitors. Another interesting speculation involves Tumor Microenvironment (TME) and the mechanisms of MDM2 in various types of immune cells. In summary, combining MDM2 inhibitors with ICI therapy offers a promising strategy to enhance anti-tumor efficacy and bypass resistance, especially in cases where MDM2 monotherapy is suboptimal. Furthermore, this combined approach does not appear to heighten the toxicity associated with the MDM2 inhibitor.

##### Dual MDM2/MDMX Inhibitors

Stapled peptides are a novel therapeutic modality capable of disrupting protein–protein interactions like MDMX-p53. In particular, MDMX (also known as MDM4) can suppress p53 independently of MDM2. Dual inhibitors (e.g., ALRN-6924), used as chemoprotective agents, aim to overcome this compensatory resistance mechanism. A pre-clinical study explored the potential of ALRN-6924 as a combination therapy for hormone receptor-positive (ER+) breast cancer. ALRN-6924 was only effective in cancer cells with a functional, wild-type TP53 gene. When combined with chemotherapy drugs like paclitaxel and eribulin, ALRN-6924 showed a powerful synergistic effect, significantly increasing its anti-tumor activity both in cell cultures and in animal models. The strong synergy observed with ALRN-6924 and chemotherapy suggests that this combination warrants further investigation in clinical trials for patients with hormone receptor-positive breast cancer [30].

In a phase I clinical trial (NCT 02264613), ALRN-6924, two treatment schedules were assessed for safety, pharmacokinetics, pharmacodynamics, and antitumor efficacy in patients with solid tumors or lymphomas. ALRN-6924 resulted in well-tolerated and exhibited promising antitumor activity [31].

##### PROTACs (Proteolysis-Targeting Chimeras)

A paradigm shift in MDM2 targeting has emerged with PROTAC-based agents such as KT-253 [32], which combines an MDM2-binding warhead with an E3 ligase recruiter (i.e., CRBN) to induce proteasomal degradation of MDM2. These bifunctional molecules selectively degrade MDM2 through ubiquitin-mediated proteasomal pathways, reducing MDM2 concentration. Preclinical studies suggest greater efficacy with advantages in resistance settings and reduced toxicity compared to traditional inhibitors [33].

Furthermore, next-generation compounds like KT-253 and the dual MDM2/MDMX inhibitor ALRN-6924 challenge conventional drug design through their beyond Rule of Five (bRo5) properties, including high molecular weight and extended surface engagement [34]. Although these features complicate pharmacokinetics, they enable unprecedented specificity for MDM2’s shallow interaction surface.

##### Chimeric Antigen Receptor T

The integration of MDM2 inhibitors with Chimeric Antigen Receptor (CAR) T cell therapy is gaining significant research focus due to the potential for synergistic effects. While CAR T cells have achieved remarkable success in certain hematologic malignancies, their widespread use, particularly in solid tumors, is often hindered by the immunosuppressive tumor microenvironment (TME) and poor T cell persistence. Modulation of p53 through MDM2 inhibition can address these limitations by enhancing CAR T cell efficacy and specificity, given p53’s central role in regulating apoptosis and the cell cycle. Furthermore, p53 activity is crucial as it influences immune responses, including immune checkpoint regulation (like PD-L1) and cytokine production, essential factors for sustained CAR T cell activation. Mechanistically, the pharmacological activation of p53, achievable with MDM2 inhibitors, has been shown to induce ligands such as ULBP1 and ULBP2 on cancer cells, promoting NKG2D-dependent cytotoxicity by lymphocytes and providing a clear rationale for combining these inhibitors with NKG2D-redirected CAR-T products [35].

##### MAPK Pathway Cross-Talk

Targeting the crosstalk between MDM2 and the RAS/MAPK pathway represents a compelling therapeutic strategy to overcome drug resistance. It has been reported that MDM2 antagonists can induce a paradoxical activation of ERK1/2 via a p53-dependent feedback loop involves mitochondrial p53 translocation and ROS-mediated activation of Receptor Tyrosine Kinases (RTKs). This compensatory activation of MAPK signaling limits the efficacy of MDM2 inhibitors as monotherapy. Consequently, combinatorial approaches targeting both MDM2 and the MAPK pathway (e.g., MEK inhibitors or RTK inhibitors) have demonstrated synergistic antitumor activity in preclinical models of dedifferentiated liposarcoma and lung adenocarcinoma, preventing the onset of resistance and promoting prolonged tumor control [36,37].

#### 2.6.2. Novel Approaches: Ongoing Clinical Trials of MDM2 Inhibitors

In addition to completed studies, several ongoing clinical trials aim to define the therapeutic window and expand the applicability of MDM2 inhibitors:

##### Siremadlin (HDM201)

A Phase Ib/II trial (NCT05447663) is evaluating siremadlin in AML patients post-allogeneic stem cell transplant to prevent relapse. The study investigates both monotherapy and combination with donor lymphocyte infusions [38].

##### Alrizomadlin (APG-115)

A phase I trial (CTR20170975) is evaluating safety, pharmacological profiles and preliminary antitumor activity of Alrizomadlin in patients with advanced solid tumors including liposarcoma (LPS) [26]. As a potent and selective oral antagonist, Alrizomadlin works by destabilizing the p53–MDM2 protein interaction, which in turn promotes p53 activation. The data suggest that Alrizomadlin, a new MDM2/p53 inhibitor, is a promising candidate deserving of further study.

##### Milademetan (DS-3032b)

A Phase I/II trial (NCT03634228) assesses the combination of milademetan with low-dose cytarabine, with or without venetoclax, in newly diagnosed or relapsed/refractory AML patients. Primary endpoints include tolerability and response rates. Subsequently, several pre-clinical trials supported a phase II (NCT05012397) basket study (MANTRA-2) in patients with advanced MDM2amp, TP53-wt solid tumors demonstrating a manageable safety profile and achieved responses against a variety of refractory MDM2amp, TP53-wt solid tumors, but tumor reductions were short-lived [25]. Another first-in-human phase I clinical trial (NCT01877382) was designed to evaluate the safety and efficacy of milademetan in patients with advanced liposarcoma, solid tumors, or lymphomas. The study concluded that intermittent dosing could reduce hematologic complications while preserving efficacy, leading to plans for a randomized Phase III trial (MANTRA) [26].

##### Idasanutlin + Venetoclax

Another Phase Ib/II study (NCT03850535) investigates the dual targeting of MDM2 and BCL-2 pathways using idasanutlin and venetoclax in relapsed/refractory AML [39]. This approach aims to enhance mitochondrial apoptosis.

**Figure 2:**
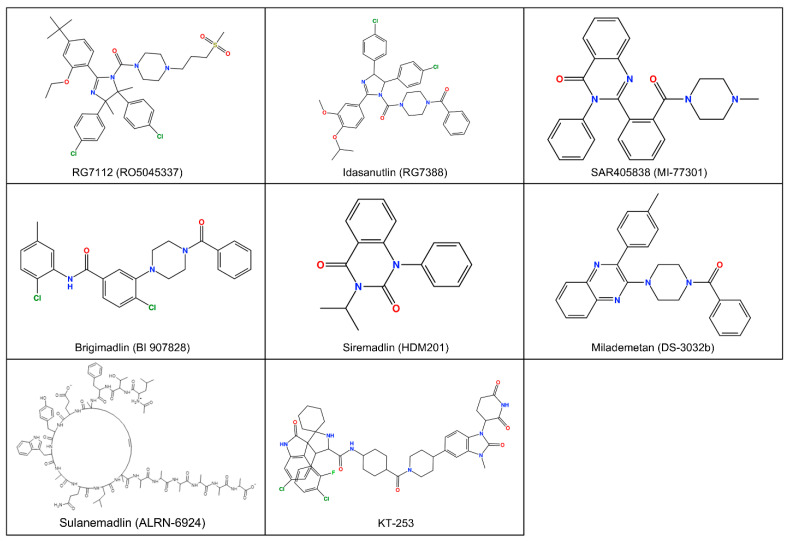
Chemical 2D structure of compounds targeting MDM2 in clinical phase.

Table 2 NCT03634228; NCT03850535, CTR20170975, NCT05012397, NCT01877382) [20,21,26,35,39] reflect ongoing interest in exploring a potential synergistic effect combining MDM2 inhibition with other modalities to enhance therapeutic efficacy and overcome resistance, particularly in hematologic malignancies. Please see Figure 2 for MDM2 inhibitors structure.

### 2.7. Clinical Implications of MDM2 Inhibition 

MDM2-targeted therapy has shown encouraging potential in several tumor types—particularly those with wild-type TP53 and MDM2 overexpression or amplification. Below are key malignancies where MDM2 inhibition is being actively explored:

#### 2.7.1. Acute Myeloid Leukemia

In Acute Myeloid Leukemia (AML), MDM2 is highly significant due to its critical role in regulating the p53 tumor suppressor protein. In particular, in cancers with wild-type TP53 (TP53-WT), like 80% of patients with AML, targeting MDM2 may be a promising therapeutic strategy. The MIRRORS clinical trial (NCT02545283) is a randomized, placebo controlled, phase III trial to evaluate the combination between cytarabine ± idasanutlin in relapsed or refractory AML [20]. Another interesting strategy was evaluated testing dual MDMX/MDM2 inhibitors like ALRN-6924, recently entered phase I clinical testing. This molecule has been demonstrated to inhibit cellular proliferation by inducing cell cycle and arrest and apoptosis in cell lines and primary AML patient cells [40].

#### 2.7.2. Dedifferentiated Liposarcoma (DDLPS)

DDLPS is the paradigmatic example of an MDM2-driven tumor. Over 90% of DDLPS tumors harbor amplification of the MDM2 gene, making it both a diagnostic hallmark and a prime therapeutic target [16].

The Brightline-1 trial is a pivotal Phase II/III study evaluating brigimadlin (BI 907828) versus doxorubicin as first-line therapy in patients with advanced or metastatic DDLPS. The study includes a crossover design and incorporates quality-of-life endpoints (e.g., EORTC QLQ-C30, PGIS/PGIC) to assess both efficacy and patient experience [16]. Preliminary data suggest that brigimadlin offers improved disease control and a favorable safety profile.

#### 2.7.3. Urothelial Carcinoma (UC)

In muscle-invasive and metastatic UC, MDM2 overexpression has been linked to resistance to platinum-based chemotherapy, immune checkpoint inhibitors (ICIs), and immunotherapy. Molecular profiling shows that MDM2-amplified tumors exhibit lower CD8 + T-cell infiltration and decreased PD-L1 expression, contributing to an immune-cold microenvironment. These findings support trials combining MDM2 inhibitors with ICIs to reprogram the tumor immune phenotype [41]. Therefore, it has been suggested that MDM2, beyond PD-L1, should be evaluated to predict a better response to combo/single therapies [42].

#### 2.7.4. Colorectal Cancer (CRC)

MDM2 amplification in CRC has been more frequently observed in primary, non-metastatic tumors than in distant metastases, suggesting a potential role in early tumorigenesis or local invasion rather than distant spread [43]. While not yet a major therapeutic target in CRC, MDM2 may gain relevance as a predictive biomarker in select subsets with intact TP53.

#### 2.7.5. Melanoma and Regional Chemotherapy Resistance

Although MDM2 overexpression is predominantly associated with systemic resistance mechanisms, emerging evidence suggests its potential role in predicting locoregional treatment outcomes. A study by Russano et al. (2023) [44] analyzed MDM2 and SURVIVIN expression in 62 patients undergoing isolated limb perfusion (ILP) with TNF and melphalan for in-transit cutaneous melanoma metastases. The results showed that high MDM2 expression was independently associated with lower rates of complete clinical response and shorter disease-free survival, suggesting a role for MDM2 in mediating resistance to TNF-based regional chemotherapy. Interestingly, patients with dual expression of MDM2 and SURVIVIN had poor outcomes, whereas those with no expression of either biomarker showed the highest rates of durable response. These findings support the potential utility of MDM2 expression as a predictive biomarker even in locoregional therapeutic settings and warrant further exploration of MDM2-targeted agents to enhance ILP efficacy in melanoma [44].

#### 2.7.6. Glioblastoma

The strategy of inhibiting the interaction between MDM2 and p53 is a promising new approach to fight brain cancer, especially in the treatment of glioblastoma (GBM). Preclinical studies in cell cultures and animal models have shown encouraging results, suggesting that MDM2 inhibitors may be effective for a specific group of GBM patients who have a wild-type (non-mutated) TP53 gene [45]. In addition, the idea is that combining MDM2 inhibitors with standard treatments like radiation and chemotherapy, or with other targeted molecular therapies, could enhance the overall effect and lead to a more significant therapeutic response also in GBM.

Despite these promising findings, many MDM2 inhibitors are still in the early stages of clinical trials for GBM. Their efficacy, both alone and in combination, has not yet been definitively confirmed [46].

#### 2.7.7. Breast Cancer

MDM2 role in breast cancer is complex and involves both p53-dependent and p53-independent mechanisms. MDM2 levels can be elevated in up to 40% of estrogen receptor-positive breast cancers, often due to gene amplification or overexpression [47]. Breast cancer cells with estrogen and progesterone receptors showed MDM2 overexpression in wild-type TP53. MDM2 treatment is specific to breast cancer patients with wild-type TP53, because of the modified binding of TP53 to MDM2. In patients with breast cancer in the advanced stages of metastasis, SMAD family member 3 (SMAD3) has been implicated in inducing MDM2 transcription via its second promoter. MDM2 expression may provide a more accurate prognostic indicator in breast cancer patients than solely evaluating the p53 status. As discussed in the previous cases, a combination therapy approach appears to be the most promising therapeutic alternative also for breast cancer [47,48].

### 2.8. Future Perspectives

Several avenues are being investigated to enhance the clinical effectiveness of MDM2 inhibitors.

Biomarker Stratification: TP53 mutational status remains the primary criterion for patient selection. However, additional markers (such as MDMX co-expression, p14ARF levels, and immune profiles) may refine patient stratification, thus optimizing the identification of those most likely to benefit from treatment [11,31].

Overcoming Resistance: The emergence of resistance to MDM2 inhibitors is a major limit to their long-term effectiveness. The most common causes include acquisitive mutations in the TP53 gene that compromise its function, overexpression of MDMX, compensatory for MDM2 inhibition, and activation of alternative pro-oncogenic signalling pathways such as PI3K/AKT and MAPK. For this reason, combination therapy strategies aimed at bypassing or overcoming these resistance mechanisms are currently being studied. These approaches include the use of dual inhibitors that act simultaneously on MDM2 and MDMX, combination with immunotherapies based on immune checkpoint blockade (e.g., anti-PD-1/PD-L1, anti-CTLA-4), and the use of PROTACs for the targeted degradation of MDM2, potentially overcoming the limitations of traditional inhibitors [31,32,49].

Toxicity Management: Hematologic toxicities, including thrombocytopenia and neutropenia, are common effects of MDM2 inhibitors. This often limits the dose that can be administered and compromises the treatment compliance. In the MANTRA-2 study [26] evaluating Milademetan, toxicity management required frequent dose interruptions (occurring in 28 of 40 patients), confirming that dosing modulation is the primary clinical tool. Similarly, in the Phase III MIRROS trial [20] for Idasanutlin, despite mandatory prophylaxis (antibiotics/antifungals), rates of febrile neutropenia remained high (52.8% in the treatment arm). Notably, the MIRROS trial data suggested that standard supportive care has limitations in this specific context; neutrophil recovery time was not significantly improved by G-CSF administration (median 36 days with G-CSF vs. 37.5 days without). Optimizing dosing schedules, including intermittent or fractionated administration and using supportive care interventions (e.g., GCSFs, transfusions) are crucial to sustaining the therapeutic index [20]. The Milademetan study explicitly notes that previous continuous low-dose strategies resulted in high discontinuation rates due to cytopenias. Consequently, newer trials have adopted intermittent high-dose strategies (e.g., Milademetan given days 1–3 and 15–17 every 28 days) to maximize tumor apoptosis while providing a “drug-free” window to allow normal hematopoietic progenitors to recover. This trend is consistent across agents; for example, Alrizomadlin was administered on an intermittent schedule (every other day for 21 days, followed by 7 days off) to mitigate bone marrow toxicity given its short half-life. We have emphasized that while an intermittent schedule improves tolerability, the therapeutic window remains narrow, as Grade 3/4 toxicities are still observed even with these optimized regimens.

Novel Delivery Platforms: Nanocarrier formulations and tumor-selective delivery mechanisms would allow overcoming the limitations associated with systemic distribution, enhancing issue-specific activity and reducing off-target toxic effects. These new strategies are under evaluation in different preclinical settings.

Expanding to New Indications: Beyond DDLPS and AML, MDM2 inhibitors are being investigated in breast cancer, NSCLC, and gliomas with wild-type TP53 [32,50,51].

## 3. Conclusions

MDM2 is a critical negative regulator of p53 and functions as an oncogenic driver in a wide array of human cancers. Its overexpression or amplification contributes to tumorigenesis via both p53-dependent and independent mechanisms. The therapeutic strategy of disrupting the MDM2-p53 interaction has led to the development of multiple targeted agents, some of which have shown encouraging results in preclinical and early clinical trials, particularly in tumors with wild-type TP53, such as dedifferentiated liposarcoma and certain hematologic malignancies. However, the clinical utility of MDM2 inhibitors is tempered by challenges such as acquired resistance (e.g., TP53 mutations), MDMX co-overexpression, and hematologic toxicity.

Rational combination strategies, including association with chemotherapy, immune checkpoint inhibitors (to enhance tumor immunogenicity and bypass immune evasion), and dual degraders or PROTACs (to selectively degrade MDM2 and potentially overcome resistance and toxicity), may improve the efficacy and durability of response to MDM2 inhibitors.

Ongoing later-stage clinical trials such as MIRROS (Idasanutlin + cytarabine in AML) and Brightline-1 (Brigimadlin vs. doxorubicin in DDLPS) are crucial for definitively establishing the role and clinical impact of these molecules. Future perspectives focus on optimizing dosing schedules to manage hematologic toxicities, utilizing additional biomarkers (beyond TP53 mutational status) for better patient stratification, and expanding their use to new indications like breast cancer, non-small-cell lung cancer (NSCLC), and gliomas, all harboring wild-type TP53 status. In particular, for concerns regarding hematological toxicities, a strict hematologic monitoring and schedule adaptation are the most effective mitigation strategies. The toxicity is “on-target” precisely because hematopoietic stem cells (HSCs) and progenitors are TP53 wild-type and actively cycling. The inhibition of MDM2 causes acute accumulation of p53 in healthy progenitors. This triggers:

- Cell cycle arrest and apoptosis: Activated p53 induces p21 and PUMA expression, halting progenitor proliferation.
- Specific defect in megakaryopoiesis: As noted in the Alrizomadlin and Milademetan studies, thrombocytopenia is a major challenge because p53 activation specifically inhibits megakaryocyte differentiation and survival.

The vulnerability stems from the fact that healthy HSCs rely on fine-tuned p53 oscillation for homeostasis. Pharmacologic hyperactivation disrupts this balance, causing transient cytopenia. This differs from the desired irreversible apoptosis in tumor cells but necessitates the “recovery periods” discussed in the dosing section.

Ongoing trials will further clarify the role of MDM2 inhibition across solid and hematologic malignancies. In summary, MDM2 represents both a robust biomarker and a therapeutically actionable target for future precision oncology.

## Figures and Tables

**Table 1:** Results of the principal MDM2-inhibitor clinical trial.

Trial (Drug/Regimen)	ORR (Objective Response Rate)	PFS (Progression-Free Survival) or Equivalent	Hematologic Adverse Events Grade 3–4 (Incidence)
MIRROS (Phase 3)	ORR: 38.8% Placebo-C: 22.0%	Median Event-Free Survival (EFS): Idasa-C: 6.3 weeks Placebo-C: 4.4 weeks	• Febrile Neutropenia: Idasa-C: 52.5%; Placebo-C: 49.3% • Thrombocytopenia: Idasa-C: 40.8%; Placebo-C: 47.9% • Anemia: Idasa-C: 23.2%; Placebo-C: 28.1%
MANTRA-2 (Phase 2)	ORR: 3.2% (1/31) (95% CI, 0.1–16.7%),	Median PFS: 3.5 months (95% CI, 1.8–3.7 months),	• Thrombocytopenia: 25.0% • Neutropenia: 17.5% • Anemia: 12.5% • Leukopenia: 10.0%
Alrizomadlin (Phase 1)	ORR: 10.0% (2/20) (95% CI, 1.2% to 31.7%),	Median PFS: 6.1 months (95% CI, 1.7–10.4 months),	• Lymphocytopenia: 33.3% • Thrombocytopenia: 33.3% • Neutropenia: 23.8% • Anemia: 23.8% • Leukopenia: 23.8% • Febrile Neutropenia: 9.5%

**Table 2:** Compounds targeting MDM2 in clinical phase.

Compound Name	Clinical Phase	Study ID
Nutlin-3a (RG7112)	Phase I	NCT00623870, NCT00559533
Idasanutlin (RG7388)	Phase III	NCT02545283 (MIRROS), NCT02624986, NCT03566485
MI-77301 (SAR405838)	Phase I	NCT01636479
AMG-232 (Navtemadlin, KRT-232)	Phase I–III	NCT01723020, NCT03662126, NCT04116541
DS-3032b (Milademetan)	Phase I/II	NCT01877382, NCT02343172, NCT05012397
BI 907828 (Brigimadlin)	Phase I–III	NCT03449381 (Brightline-2), NCT06058793 (Brightline-4)
APG-115 (Alrizomadlin)	Phase I/II	NCT02935907, NCT03781986, NCT04785196, CTR20170975
HDM201 (Siremadlin)	Phase I/II	NCT02143635, NCT03107780
ALRN-6924 (Sulanemadlin)	Phase I/II	NCT02264613
KT-253	Phase I (ongoing)	NCT05775406

## Data Availability

No new data were created or analyzed in this study. Data sharing is not applicable to this article.

## Abbreviations

The following abbreviations are used in this manuscript:

BTC	Biliary tract cancer
CRC	Colorectal Cancer
DDLPS	Differentiated Liposarcoma
ICIs	Immune Checkpoint Inhibitors
ILP	Isolated Limb Perfusion
MDM2	Murine Double Minute 2
NSCLC	Non-Small-Cell Lung Cancer
PDAC	Pancreatic ductal adenocarcinoma
PPI	Protein–Protein Interaction
PROTACs	Proteolysis-Targeting Chimeras
RITA	Reactivation of p53 and Induction of Tumor cell Apoptosis
UC	Urothelial Carcinoma
